# Role of glucose metabolism related gene GLUT1 in the occurrence and prognosis of colorectal cancer

**DOI:** 10.18632/oncotarget.18090

**Published:** 2017-05-23

**Authors:** Wenming Feng, Ge Cui, Cheng-Wu Tang, Xiao-Lan Zhang, Chuang Dai, Yong-Qiang Xu, Hui Gong, Tao Xue, Hui-Hui Guo, Ying Bao

**Affiliations:** ^1^ Department of Hepatobiliary Pancreatic Surgery, The First Affiliated Hospital of Huzhou University, Huzhou, Zhejiang Province, P.R. China; ^2^ Department of Pathology, The First Affiliated Hospital of Huzhou University, Huzhou, Zhejiang Province, P.R. China; ^3^ Department of Surgery, The First Affiliated Hospital of Huzhou University, Huzhou, Zhejiang Province, P.R. China; ^4^ Central Laboratory, The First Affiliated Hospital of Huzhou University, Huzhou, Zhejiang Province, P.R. China

**Keywords:** colorectal cancer, GLUT1, polymorphism, prognosis

## Abstract

Colorectal cancer (CRC) ranks the third most commonly diagnosed cancer in males and the second in females worldwide. However, the functional and causal SNPs for CRC remain to be mined. Glucose transporter 1 (GLUT1), a pivotal rate-limiting element in the transport of glucose in malignancy cells, has been identified to be associated with many cancers. Here, we aim to explore the role of GLUT1 in the occurrence and prognosis of colorectal cancer in a Chinese population. We found that GLUT1 expression levels in CRC tumor tissues were significantly higher than those in the corresponding adjacent normal tissues, and Cox multivariate analysis demonstrated that the GLUT1 expression was an independent prognostic factor for CRC (HR = 2.11, 95% CI = 1.33–3.34, P=0.001). For a functional polymorphism of GLUT1 (rs710218), we found that individuals with TT genotype (OR = 1.68, 95% CI = 1.02-2.75, P = 0.041) or AT genotype (OR = 1.47, 95% CI = 1.09-1.99, P = 0.012) of rs710218 had a significantly increased risk of CRC compared to those with AA homozygote. These findings strongly suggest that glucose metabolism related gene GLUT1, and its functional SNP, rs710218 might contribute to CRC susceptibility and prognosis, and the exact biological mechanism awaits further research.

## INTRODUCTION

Colorectal cancer (CRC) ranks the third most commonly diagnosed cancer in males and the second in females worldwide [1]. According to newly published “Cancer Statistics, 2017” of United States, the estimated new CRC cases were 135,430, and the new deaths were 50,260 in 2017 [2]. In china, the annual CRC cases were 376.3 thousands, while the deaths were 191.0 thousands [3]. CRC has become a major public health problem. It is well established that CRC is a complex trait caused by genetic and environmental factors and their interactions [4–6]. Genome-wide association studies (GWASs) have identified numerous susceptibility loci for CRC, however, most risk variants are located in non-coding regions without clear biological mechanisms [7–13]. Thus, the functional and causal SNPs for CRC remain to be mined.

Glucose transporter 1 (GLUT1), also named facilitates glucose transporter member 1 (SLC2A1), has been demonstrated to be a pivotal rate-limiting element in the transport of glucose in malignancy cells and overexpressed in different types of human cancers [14–19]. Oh et al. [20] also found that glut1 could promote cell proliferation, migration and invasion by regulating epidermal growth factor receptor and integrin signaling in triple-negative breast cancer cells. It was also a prognostic molecular biomarkers for patients with colorectal cancer liver metastasis [21]. Recently, a meta-analysis also revealed that the expression status of GLUT1 was a vital prognostic indicator and promising therapeutic target in solid tumors [22]. A functional polymorphism (GLUT1 rs710218), which is localized in the promoter region and 2841 bp upstream of the start of exon 1 of GLUT1, consists of an A to T substitution and is closely positioned to a number of putative binding sites for transcription factors, including HIF-1alpha [23]. Previous studies have been conducted to evaluated associations between SNP rs710218 and susceptibility of hepatocellular carcinoma, diabetic nephropathy, breast cancer, in-stent restenosis, and clear-cell renal carcinoma [23–27]. Here, we aim to explore the role of glucose metabolism related gene GLUT1 in the occurrence and prognosis of colorectal cancer in a Chinese population.

## RESULTS

### Characteristics of study populations

The characteristics of the study participants were summarized in Table 1. Totally 368 CRC cases and 500 healthy controls were included in this study. No significant differences were found between cases and controls for age, gender, smoking status, and drinking status. However, CRC cases have higher Body mass index and Waist-hip-ratio than healthy controls (P<0.001). More tumors were located at Colon, while TNM stage II and III account for 76.4% of all CRC cases.

**Table 1:** The characteristics of the study population

Variables	Cases (n=368)	Controls (n=500)	P value
Age			
	52.8±5.5	52.9±5.6	0.791
Gender			
Male	213	285	0.795
Female	155	215	
Smoking status			
Smokers	125	141	0.068
Non-smokers	243	359	
Alcohol status			
Drinkers	139	179	0.551
Non-drinkers	229	321	
Body mass index			
	24.2±2.5	23.1±2.4	**P<0.001**
Waist-hip-ratio			
	0.82±0.004	0.81±0.005	**P<0.001**
Tumor site			
Colon	207		
Rectum	161		
TNM stage			
I	60		
II	114		
III	167		
IV	27		

### GLUT1 expression and CRC susceptibility

**Figure 1:**
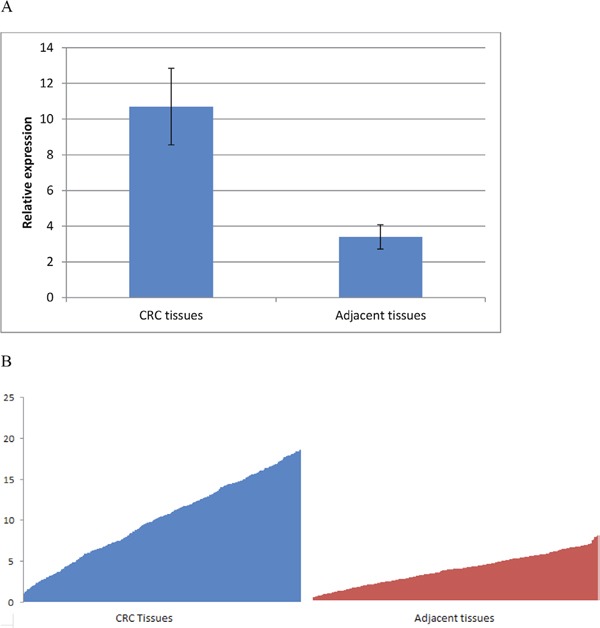
GLUT1 expression is up-regulated in CRC tissues **(A)** Mean value ± SD and **(B)** inter-individual variability in gene expression levels.

Firstly, we examined GLUT1 level in CRC tumor tissues. The expression of GLUT1 in tumor tissues relative to adjacent normal tissues is shown in Figure 1. Among all the 368 pairs of CRC patients, GLUT1 expression levels in CRC tumor tissues were significantly higher than those in the corresponding adjacent normal tissues (P<0.001), which indicates that GLUT1 contributes to the susceptibility of CRC.

### GLUT1 predicts poor prognosis in CRC patients

**Figure 2:**
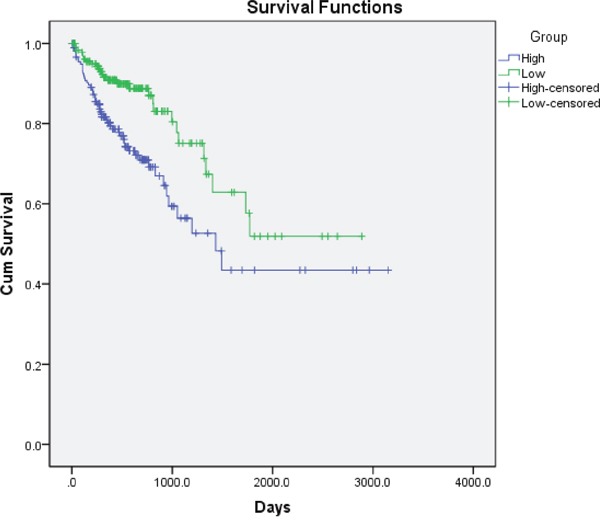
Kaplan–Meier overall survival curves for 368 CRC patients classified according to relative GLUT1 expression levels in CRC tissues

In order to further evaluate the value of GLUT1 in prognosis of patients with CRC, we used Kaplan–Meier survival analysis and log-rank test. These 368 CRC patients were classified into relatively high or low GLUT1 expression group using the median level of GLUT1 in CRC tissues as a cut-off value. As shown in Figure 2, the increased expression of GLUT1 was significantly associated with poor survival of CRC (P=0.001). After adjusting for age, gender, smoking status, drinking status, body mass index, and waist-hip-ratio, Cox multivariate analysis demonstrated that the GLUT1 expression was an independent prognostic factor for CRC (HR = 2.11, 95% CI = 1.33–3.34, P=0.001).

### Association analysis between individual SNP and CRC risk

**Figure 3:**
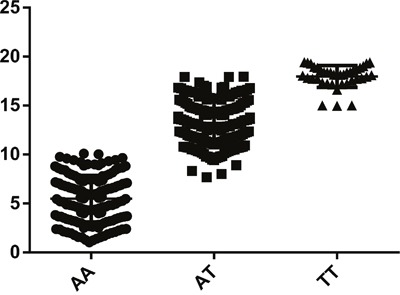
Comparison between rs710218 and GLUT1 expression

As shown in Table 2, GLUT1 rs710218 was evidently associated with increased CRC risk. Under multivariable logistic regression model adjusted for age, gender, smoking status, drinking status, body mass index, and waist-hip-ratio, individuals with TT genotype (OR = 1.68, 95% CI = 1.02-2.75, P = 0.041) or AT genotype (OR = 1.47, 95% CI = 1.09-1.99, P = 0.012) of rs710218 had a significantly increased risk of CRC compared to those with AA homozygote. In dominant model, TT genotype was significant associated with increased CRC risk (OR = 1.51, 95% CI = 1.13-2.00, P = 0.005). Likewise, positive outcome was found in the additive models, with per-T-allele OR of 1.37 (95% CI = 1.09-1.71, P = 0.006). Results of Figure 3, which presents comparison between rs710218 and GLUT1 expression, also indicated that T allele was significantly associated with higher GLUT1 expression (P<0.001) and then contributed to CRC risk.

**Table 2:** Associations of GLUT1 rs710218 with CRC risk

	CRC cases	Controls	OR (95% CIs) *	P value
AA	156	258	1.00 (Reference)	
AT	173	202	1.47 (1.09-1.99)	**0.012**
TT	39	40	1.68 (1.02-2.75)	**0.041**
T vs A			1.37 (1.09-1.71)	**0.006**
TT+AT vs AA	212/156	242/258	1.51 (1.13-2.00)	**0.005**
TT vs AT+AA	39/329	40/460	1.42 (.086-2.33)	0.168

* Adjusted for age, gender, smoking status, drinking status, body mass index, and waist-hip-ratio.

## DISCUSSION

The current study explored association between glucose metabolism related gene GLUT1 and the occurrence and prognosis of colorectal cancer in a Chinese population; and it also evaluated a functional SNP of GLUT1, rs710218 might contribute to CRC susceptibility. To be best of our knowledge, this should be the first study aiming to evaluate association of the expression of GLUT1 and CRC in Chinese population, and also should be the first genetic association study about polymorphism of GLUT1 and CRC risk.

Glucose metabolism was closely related with carcinogenesis of CRC [28–31]. Early in 1981, Ingram et al. [32] has reported that glucose could increase experimentally induced colorectal cancer. Since, many experimental and epidemiological studies have concluded their relationship [31, 33–38]. In 1999, In the Cardiovascular Health Study cohort of 5849 participants, Schoen et al. [35] found that Individuals in the highest quartile of fasting glucose had a nearly twofold increased risk of CRC, and the linear trend RR for fasting glucose level was statistically significant. That should be the first direct evidence of an association between elevated visceral adipose tissue level, its associated metabolic effects, and colorectal cancer [35]. GLUT1, a uniporter protein encoded by the SLC2A1 gene in humans, is in charge of facilitating the transport of glucose across the plasma membranes of mammalian cells [39]. The expression levels of GLUT1 in cell membranes could be increased by reduced glucose levels and decreased by increased glucose levels [40]. Recent study showed that elevated expression of TrpC5 and GLUT1 is associated with chemo-resistance in CRC patients [41]. In current study, we found that GLUT1 expression levels in CRC tumor tissues were significantly higher than those in the corresponding adjacent normal tissues, and Cox multivariate analysis demonstrated that the GLUT1 expression was an independent prognostic factor for CRC. These results were consistent with the finding the meta-analysis by Wang et al. [22], although no studies have evaluated the association between GLUT1 expression and CRC prognosis in Chinese population.

SNP rs710218 was located in the promoter region of the GLUT1 gene (-2841) closely positioned to a hypoxic response element (HRE) as putative HIF-1alpha binding site [24]. Page et al. [27] found that there was a highly significant decrease in the A-2841 genotype (P=0.0004) in the promoter region of those patients with CCRCC compared to the control population, while Thomas et al. [26] identified that rs710218 is not associated with a higher risk for HCC but rather for HCC progression. In current study, our findings, that rs710218 was significantly associated with increased risk of CRC, were consistent with conclusion of Page et al. [27]. Further replication in different population and ethnics are warranted.

In conclusion, our results indicate that the expression status of GLUT1 in CRC tumor tissues were significantly higher than those in the corresponding adjacent normal tissues, and increased expression of GLUT1 was significantly associated with poor survival of CR. These results made it could be used as a promising biomarker of occurrence and unfavorable prognosis for CRC. We also found SNP rs710218, a functional variant in the promoter region of the GLUT1 gene, was significantly associated with increased risk of CRC. Further exploration on its function is needed to clarify the biological mechanism behind.

## PATIENTS AND METHODS

### Study subjects

A total of 868 participants were enrolled in the present study. The case group consisted of 368 clinically and pathologically confirmed CRC patients, and 500 healthy checkup individuals were selected as the controls, which were matched by age, and gender. Demographic data, such as individual's age, gender, smoking, drinking, anthropometric measures, and pathological features, were collected by a face to face interview. Meanwhile, 5 mL peripheral blood from each individual was collected for genomic DNA extraction, and tissues were immediately snap-frozen in liquid nitrogen and stored at −80°C until total RNA was extracted for gene expression assay. The study was approved by Research Ethics Committee of the hospital, and written informed consent was obtained from all patients.

### Tissue RNA extraction and quantitative RT-PCR analysis

Total RNA from frozen tissues were isolated with TRIzol reagent (Invitrogen, Carlsbad, CA, USA) according to the manufacturer's protocol. The quality of the RNA samples were measured using Agilent 2100 bioanalyzer. Only samples that have an Integrity Number value above 6.0 that indicate acceptable RNA integrity for RT-PCR assays can be included in the study. Or the RNA would be extracted again. Then, RNA was reverse transcribed to cDNA by using a PrimeScript RT reagent Kit (Takara, Dalian, China). The SYBR Premix Ex Taq (Takara, Dalian, China) was used to detect GLUT1 expression according to the manufacturer's instructions. Real-time PCR and data collection were performed on ABI 7500. We have searched for a most suitable reference gene using NormFinder, which indicated GAPDH was suitable. Then the relative gene expression levels of GLUT1 were normalized to the expression of GAPDH, and calculated utilizing the 2^−ΔCt^ method.

### DNA extraction and genotyping

Genomic DNA specimens were prepared from 200 μL blood using the QIAamp blood kit following the manufacturer's instructions (Qiagen, Hilden, Germany). The genotype analysis was performed using SEQUENOM's MassARRAY iPLEX assay according to the instructions of the manufacturer. For quality control, To validate the genotyping method, we also analyzed 5% randomly selected DNA samples by direct sequencing; the results for these 2 methods were 100% concordant. Also, approximately 5% of the total DNA samples were randomly selected for genotyping in duplicate by two independent technicians to confirm a 100% consistency.

### Statistical analysis

Demographic difference for the patients and controls were compared using Student's t-test and the chi-square test for the continuous variables and categorical variables, respectively. The Hardy-Weinberg equilibrium (HWE) for each candidate variant was assessed by a goodness-of-fit χ2 test in the control group. The associations of genetic factors and CRC risks were estimated by logistic regression model with adjustment for age, gender, smoking status, drinking status, body mass index, and waist-hip-ratio. The survival curves were estimated using the Kaplan–Meier method. The log-rank test was used to estimate the significance of the differences between the survival curves. A Cox proportional hazards analysis was performed to calculate the hazard ratio (HR) and the 95 % confidence interval (CI) to evaluate the association between GLUT1 expression and overall survival time (OS). All statistical analyses were performed with SPSS 13.0 (IBM Corporation, Armonk, NY) and all P values were two sided with P< 0.05 considered significant.
